# Clinical Utility of the Addition of Molecular Genetic Testing to Newborn Screening for Sickle Cell Anemia

**DOI:** 10.3389/fmed.2021.734305

**Published:** 2021-11-10

**Authors:** Lisa M. Shook, Deidra Haygood, Charles T. Quinn

**Affiliations:** ^1^Division of Hematology, Cincinnati Children's Hospital Medical Center, Cincinnati, OH, United States; ^2^Department of Pediatrics, University of Cincinnati College of Medicine, Cincinnati, OH, United States; ^3^Erythrocyte Diagnostic Laboratory, Cincinnati Children's Hospital Medical Center, Cincinnati, OH, United States

**Keywords:** hemoglobinopathies, newborn screening, sickle cell anemia, electrophoresis, genetic testing, HPFH, hydroxyurea

## Abstract

Sickle cell disease (SCD) is a group of related yet genetically complex hemoglobinopathies. Universal newborn screening (NBS) for SCD is performed in the United States and many other nations. Classical, protein-based laboratory methods are often adequate for the diagnosis of SCD but have specific limitations in the context of NBS. A particular challenge is the differentiation of sickle cell anemia (SCA) from the benign condition, compound heterozygosity for HbS and gene-deletion hereditary persistence of fetal hemoglobin (HbS/HPFH). We describe a sequential cohort of 44 newborns identified over 4.5 years who had molecular genetic testing incorporated into NBS for presumed SCA (an “FS” pattern). The final diagnosis was something other than SCA in six newborns (12%). Three (7%) had HbS/HPFH. All had a final, correct diagnosis at the time of their first scheduled clinic visit in our center (median 8 weeks of age). None received initial counseling for an incorrect diagnosis. In summary, genetic testing as a component of NBS for SCD is necessary to provide correct genetic counseling and education for all newborns' families at their first visit to a sickle cell center. Genetic testing also permits the use of early, pre-symptomatic hydroxyurea therapy by preventing infants with HbS/HPFH from receiving unnecessary therapy. We argue that genetic testing should be incorporated into contemporary NBS for SCD.

## Introduction

Sickle cell disease (SCD) is the name for a group of related hemoglobinopathies that affects numerous populations worldwide. Although it is a monogenic disease caused by mutations of the β-globin gene (*HBB*), there are a number of distinct genotypes of SCD that require differentiation to inform clinical care. The most common and severe form of SCD is the homozygous state for the sickle hemoglobin (HbS) mutation, called sickle cell anemia (SCA) or HbSS. There are also compound heterozygous forms of SCD, such as sickle-hemoglobin C disease (HbSC), sickle-β^+^-thalassemia (HbSβ^+^), and sickle-β^0^-thalassemia (HbSβ^0^). In contrast, compound heterozygosity for the HbS mutation and gene-deletion hereditary persistence of fetal hemoglobin (HbS/HPFH) is essentially benign.

Accurate NBS for SCD is needed to permit the prompt initiation of penicillin prophylaxis and referral to a specialized SCD center for ongoing comprehensive care, which has been shown to reduce morbidity and mortality (1–4). Most NBS programs use protein-based, Hb separation techniques that include electrophoresis (gel- or liquid-based), isoelectric focusing (IEF), and high-performance liquid chromatography (5). Although protein-based methods are the mainstay of Hb diagnostics (5), they may be inadequate in the context of NBS to detect β-thalassemia mutations (6) and unable to differentiate between HbSS and HbS/HPFH (7).

Without parental studies, the clinical differentiation of HbSS from HbS/HPFH can take several years to be correctly realized, given the prolonged postnatal decline in HbF concentration in children SCD (8). In the past, a watch-and-wait approach was reasonable given the limited and usually delayed initiation of treatment options. The hazards of this approach include unnecessary venipunctures, clinic visits, and prophylactic antibiotics for the children with HbS/HPFH and anxiety and fear about a serious disease, incorrectly diagnosed, for their parents and family. Now, the increasing use of early, pre-symptomatic disease-modifying therapy (e.g., initiation of hydroxyurea by 6 months of age for SCA) has made this critical diagnostic distinction time-sensitive (9). Treating children who have HbS/HPFH with hydroxyurea is not indicated. Here we describe a sequential cohort of newborns who had a presumed diagnosis of SCA by NBS (an “FS” pattern), several of whom actually had HbS/HPFH or other forms of SCD that were correctly and rapidly diagnosed by genetic testing before the child's first visit to our center, thereby preventing incorrect counseling and unnecessary medical interventions.

## Patients and Methods

**Figure 1:**
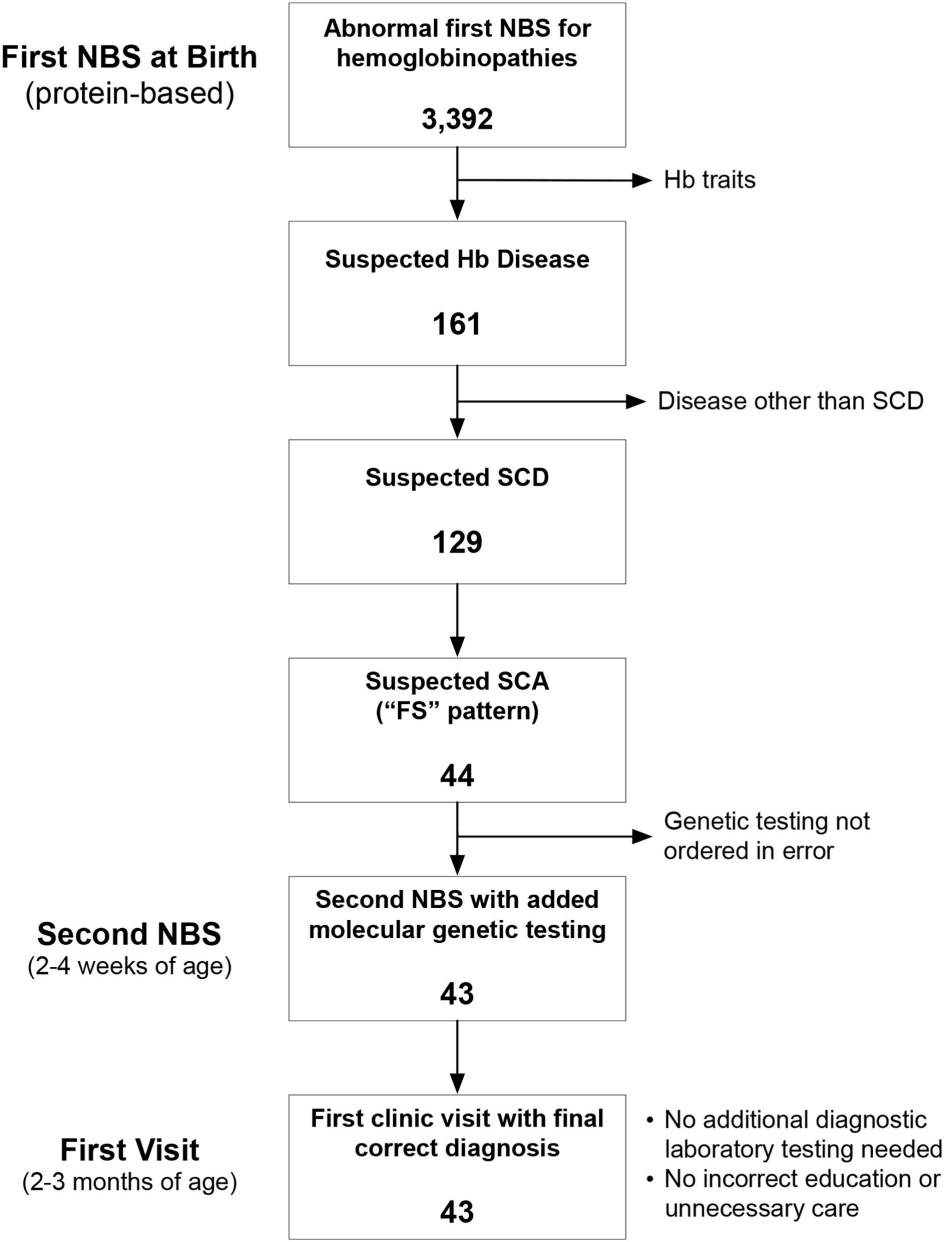
Identification of the study cohort. Between July 2015 and December 2020, there were 3,392 abnormal newborn screening (NBS) tests for hemoglobinopathies. We excluded newborns with hemoglobin (Hb) trait states and hemoglobinopathies other than sickle cell disease (SCD). We identified 44 newborns during this time with an “FS” pattern, indicating suspected sickle cell anemia (SCA), which we define here to include both homozygous sickle cell anemia (HbSS) and sickle-β^0^-thalassemia (HbSβ^0^).

Newborn screening in the state of Ohio begins with an initial dried blood spot that is analyzed at a central laboratory using a combination of HPLC and IEF. Newborns with Hb variants detected by this initial phase of testing have a second, confirmatory panel of testing performed using new blood specimens obtained by venipuncture in the first 2–4 weeks of life at a Regional Sickle Cell Services Program (RSCP). The Ohio Department of Health Region 1 RSCP is located at the Cincinnati Comprehensive Sickle Cell Center at Cincinnati Children's Hospital Medical Center (CCHMC). Region 1 comprises eight counties in southwestern Ohio in which approximately 500 newborns undergo confirmatory testing for suspected hemoglobinopathies each year. For newborns with suspected SCD, protein-based confirmatory testing includes capillary zone electrophoresis, acid hemoglobin electrophoresis, and IEF. Additionally, beginning in July 2015, newborns with an “FS” pattern indicating suspected SCA (here defined to comprise HbSS and HbSβ^0^), also had *HBB* sequence analysis and copy number variation analysis of the α-globin and β-globin gene clusters performed simultaneously using the same blood specimen. The overall goal of this NBS-based genetic testing is to differentiate HbSS from HbS/HPFH before affected newborns have their first visits in our comprehensive sickle cell center. No *a priori* sample sized was calculated, rather all consecutive patients were included in this arbitrary 4.5-year sample (Figure 1). Medians were compared by the 2-tailed Mann Whitney test. *P* < 0.05 was considered statistically significant.

## Results

We analyzed 3,392 consecutive newborns with abnormal NBS results between July 2015 and December 2020 and identified 44 who had a presumed diagnosis of SCA (Figure 1). Genetic testing was not performed for 1 newborn at the time of the second, confirmatory NBS test in error (only protein-based testing was done); genetic testing later confirmed that she had HbSS. Of the remaining 43 newborns who had genetic testing as part of NBS (Table 1), 37 (86%) had a final diagnosis of HbSS. The other six newborns (14%) had different SCD genotypes. Three (7%) had HbS/HPFH, two (5%) had sickle-β^+^-thalassemia (HbSβ^+^), and one (2%) had sickle-β^0^-thalassemia (Hb Sβ^0^).

**Table 1:** Results of molecular genetic testing as a component of newborn screening (NBS) for suspected sickle cell anemia (SCA).

**Final diagnosis (β-globin genotype)**	***N* (%)**
Sickle cell anemia (HbSS)	37 (86%)
Sickle-β^0^-thalassemia (HbSβ^0^)^a^	1 (2%)
Sickle-β^+^-thalassemia (HbSβ^+^)	2 (5%)
HbS/HPFH^b^	3 (7%)

*Forty-three consecutive newborns with an “FS” pattern on the first NBS test, indicating a likely diagnosis of SCA, had genetic testing performed as a component of the second, confirmatory NBS testing panel. In addition to protein-based testing, molecular genetic testing included direct sequencing of the β-globin gene (HBB) and copy number variation analysis of the β-globin gene cluster*.

a *HbSS and HbSβ^0^ are often both referred to as sickle cell anemia (SCA)*.

b *Compound heterozygosity for the HbS mutation and gene-deletion forms of hereditary persistence of fetal hemoglobin [(δβ)^0^-HPFH]*.

Of the newborns 3 who had HbS/HPFH, 2 had the HPFH-2 (Ghanaian) deletion and 1 had the HPFH-1 (Black) deletion. These three newborns had a final genetic diagnosis of HbS/HPFH by the time of their first clinic visits in our center (ages at first visits: 6, 8, and 11 weeks). None was started on hydroxyurea. If prophylactic penicillin was initiated before the first clinic visit it was discontinued, and each is now followed only yearly in our center. The two with HbSβ^+^ also had a final genetic diagnosis of by the time of their first clinic visit.

Of the patients with SCA, 37/38 (97.3%) were prescribed hydroxyurea starting at a median of 8.0 months of age (interquartile range: 7.1–10.0). The single patient with SCA not yet prescribed hydroxyurea was last seen in our clinic at 4 months of age, and he has a scheduled visit at 7 months of age to start this medication. Comparing the first and second halves of this cohort, the median age at prescription of hydroxyurea decreased from 9.6 to 7.8 months (*P* = 0.023).

## Discussion

In this consecutive cohort of newborns with suspected SCA, the final diagnosis was something other than HbSS or HbSβ^0^ in about 12%. Three (7%) had HbS/HPFH and 2 (5%) had HbSβ^+^. The distinction between HbSS and HbSβ^0^ by NBS is not critical, because both diseases are clinically similar and managed identically, and these two genotypes are often both called sickle cell anemia (10, 11). Nevertheless, this genetic distinction can be clinically helpful to explain microcytosis, provide accurate genetic counseling, and determine eligibility for genetic therapies.

A diagnosis of HbSβ^+^ will become clear within a few months of age by the detection of a small amount of HbA by Hb analysis, but a diagnosis of HbS/HPFH might take several years to be correctly realized. In children with SCA, the postnatal decline in HbF concentration may take 5–6 years to be completed (8). In the past, a “watch-and-wait” approach to distinguish HbSS from HbS/HPFH with serial measurements of HbF and blood counts was reasonable given the formerly limited and delayed initiation of disease-modifying therapy. Now, the increasing use of early, pre-symptomatic disease-modifying therapy (e.g., hydroxyurea initiation at 6 months of age) has made this critical diagnostic distinction time-sensitive (9, 12). For infants with SCA, hydroxyurea should be started to prevent and reverse this early decline in HbF rather than waiting for the HbF level to decline to some arbitrary level (13). However, treating children who have HbS/HPFH with hydroxyurea is not indicated and exposes them to unnecessary risks, expenses, laboratory monitoring, and inconvenience. Genetic testing incorporated into NBS can facilitate this new standard (6).

We also determine α-globin gene copy number (data not shown), because it provides supplemental prognostic information in SCA, and copy number variation analysis of the β-globin gene cluster is already being performed. We do not test for common non-gene-deletion HPFH determinants at the time of NBS, because these polymorphisms are not likely to inform early clinical management, but practice may change with the decreasing costs of broader genetic testing over time (e.g., next generation sequencing panels). Currently, the cost of genetic testing is offset by the elimination of both parental testing and additional diagnostic laboratory testing in the infant.

Molecular genetic testing is not required to determine if an infant has HbS/HPFH if parental studies can be performed. However, both parents may not be available or willing to be tested. The time needed to perform this testing may also take longer than needed to permit the earliest initiation of hydroxyurea. In this 4.5-year study period, the median age of initiation of hydroxyurea decreased from 9.6 to 7.8 months of age, highlighting the need for a timely correct diagnosis. Genetic testing added to NBS overcomes these obstacles and can be performed on a blood sample that had already been obtained for protein-based testing. A recent plea has been published for the diagnosis of HbS/HPFH in newborns (7), which we have answered here. Larger studies are needed to demonstrate the generalizability and cost-effectiveness of our approach.

In summary, molecular genetic testing incorporated into NBS for SCD allows correct education and counseling for the newborn's family to be given at the first visit in a sickle cell center and eliminates additional diagnostic testing (no additional venipuncture). It prevents unnecessary grief and anxiety from incorrectly diagnosing a newborn with SCA, only later to “revise” the diagnosis to a benign condition. Moreover, a timely correct diagnosis can prevent infants with HbS/HPFH from receiving unnecessary medical interventions and treatments.

## Data Availability Statement

The raw data supporting the conclusions of this article will be made available by the authors, without undue reservation.

## Ethics Statement

Ethical review and approval was not required for the study on human participants in accordance with the local legislation and institutional requirements. Written informed consent from the participants' legal guardian/next of kin was not required to participate in this study in accordance with the national legislation and the institutional requirements.

## Author Contributions

LS and CQ: conceptualization, visualization, and funding acquisition. CQ: formal analysis. DH: investigation and data curation. LS: writing—original draft preparation. DH and CQ: writing—review and editing. All authors have read and agreed to the published version of the manuscript.

## Funding

This research was funded in part by Ohio Department of Health, grant number 0313011SK1320 (to LS) and by NIH-NHLBI, grant number U01HL117709 (to CQ).

## Conflict of Interest

The authors declare that the research was conducted in the absence of any commercial or financial relationships that could be construed as a potential conflict of interest.

## Publisher's Note

All claims expressed in this article are solely those of the authors and do not necessarily represent those of their affiliated organizations, or those of the publisher, the editors and the reviewers. Any product that may be evaluated in this article, or claim that may be made by its manufacturer, is not guaranteed or endorsed by the publisher.
